# Electrospun Chitosan-Gelatin Biopolymer Composite Nanofibers for Horseradish Peroxidase Immobilization in a Hydrogen Peroxide Biosensor

**DOI:** 10.3390/bios7040047

**Published:** 2017-10-15

**Authors:** Siriwan Teepoo, Phanphruk Dawan, Naris Barnthip

**Affiliations:** 1 Department of Chemistry, Faculty of Science and Technology, Rajamangala University of Technology Thanyaburi, Pathumthani 12110, Thailand; p_dawan@live.com; 2 Division of Physics, Faculty of Science and Technology, Rajamangala University of Technology Thanyaburi, Pathumthani 12110, Thailand; naris.b@rmutt.ac.th

**Keywords:** nanofibers, biopolymers, nanomaterials, biosensor, electrospinning method

## Abstract

A biosensor based on chitosan-gelatin composite biopolymers nanofibers is found to be effective for the immobilization of horseradish peroxidase to detect hydrogen peroxide. The biopolymer nanofibers were fabricated by an electrospining technique. Upon optimization of synthesis parameters, biopolymers nanofibers, an average of 80 nm in diameter, were obtained and were then modified on the working electrode surface. The effects of the concentration of enzyme, pH, and concentration of the buffer and the working potential on the current response of the nanofibers-modified electrode toward hydrogen peroxide were optimized to obtain the maximal current response. The results found that horseradish peroxidase immobilization on chitosan-gelatin composite biopolymer nanofibers had advantages of fast response, excellent reproducibility, high stability, and showed a linear response to hydrogen peroxide in the concentration range from 0.1 to 1.7 mM with a detection limit of 0.05 mM and exhibited high sensitivity of 44 µA∙mM^−1∙^cm^−2^. The developed system was evaluated for analysis of disinfectant samples and showed good agreement between the results obtained by the titration method without significant differences at the 0.05 significance level. The proposed strategy based on chitosan-gelatin composite biopolymer nanofibers for the immobilization of enzymes can be extended for the development of other enzyme-based biosensors.

## 1. Introduction

Recently, biosensors have become promising devices for analytical methods in several fields, including clinical detection [1,2], the food industry [3,4], and environmental monitoring [5,6], due to their unique high sensitivity, simplicity, lack of sample preparation, and fast analysis time. A biosensor is an analytical device comprised of two components which are biological sensing elements and a transducer. When the biological elements are specifically reacted with an analyzed target, a chemical change signal is measured by a transducer [7]. Enzymes are often used as biological elements for the development of biosensors. Effective immobilization of enzyme is one of the key steps that can improve the biosensor response. In recent years, enzymes, such as horseradish peroxidase (HRP), have been immobilized on several supporting materials, including 3-aminopropyl trimethoxy silane [8], poly(aniline-co-N-methylthionine) [9], and poly(thionine) film [10]. Nanomaterials provide excellent enhancement of enzyme immobilization efficiency. Owing to their dimensions in the range of 100 nm, and the high surface-to-volume ratios provided, many have been successfully used for loading various amounts of enzymes [11]. Among the various nanomaterials, nanofibers have recently gained increased interest due to their physical and chemical properties [11,12,13]. Nanofibers have several useful properties, such as high specific surface area and high porosity [14,15]. Considerable attention has been paid to the applications of nanofibers in biosensors.

Electrospinning is the most effective method which produces fibers in the range of nanometer in diameter with high voltage electrostatic field. Electrically-charged jets were obtained when applying a high voltage to polymer solution. During the time that these jets moved toward the collector, the solvent evaporated and the nanofibers were obtained on the collector [16]. Electrospun nanofibers with small diameter, long length, large surface area per unit mass, and tunable electron transport were applied to improve the electrochemical biosensor performance [17,18]. There are a few reports on the employment of nanofibers in biosensors. Therefore, we are interested in fabricating enzymatic biosensors based on nanofibers for the detection of hydrogen peroxide using horseradish peroxidase as the biological element. In this work, chitosan-gelatin composite biopolymers were used for the fabrication of the nanofibers. Gelatin is a natural biodegradable polymer derived from collagens [19]. Nanofibers based on gelatin have been prepared for many applications [20,21]. During the past decade, chitosan has been used as a biocompatible matrix to immobilize biological sensing elements for biosensor construction [22,23]. The natural-biopolymer chitosan is a type of matrix for enzyme immobilization with excellent properties, such as good adhesion, nontoxicity, and biocompatibility [24,25]. Moreover, it was chosen for nanofiber fabrication because of its large quantities of amino groups, which have a strong binding with enzymes via covalent bonding [26]. These outstanding properties make the gelatin and chitosan biopolymers optimal candidates for nanofiber fabrication.

In the present work, we described the fabrication of novel hybrid biopolymer nanofibers for hydrogen peroxide biosensor detection. The nanofibers were characterized with scanning electron microscopy and electrochemical methods. To the best of our knowledge, this is the first fabrication of chitosan-gelatin composite biopolymer nanofibers for a hydrogen peroxide biosensor based on horseradish peroxidase. In addition, the experimental conditions for fabrication and analytical performance of the biosensor based on nanofibers were optimized. Finally, we have investigated the performance of the nanofiber biosensor, based on HRP immobilized on a chitosan-gelatin composite biopolymer nanofibers-modified electrode, for the determination of hydrogen peroxide in real samples.

## 2. Materials and Methods

### 2.1. Reagents and Materials

Horseradish peroxidase (HRP), chitosan (medium molecular weight), hydrogen peroxide, and gelatin were purchased from Sigma-Aldrich (St. Louis, MO, USA)). Acetic acid was purchased from Ajex (Taren Point, Australia). Phosphate buffer solution (0.1 M) was prepared with Na_2_HPO_4_ and NaH_2_PO_4_. All other reagents were of analytical grade. All aqueous solutions were prepared with deionized water.

### 2.2. Preparation of Gelatin/Chitosan Nanofibers by Using the Electrospinning Method

**Figure 1:**
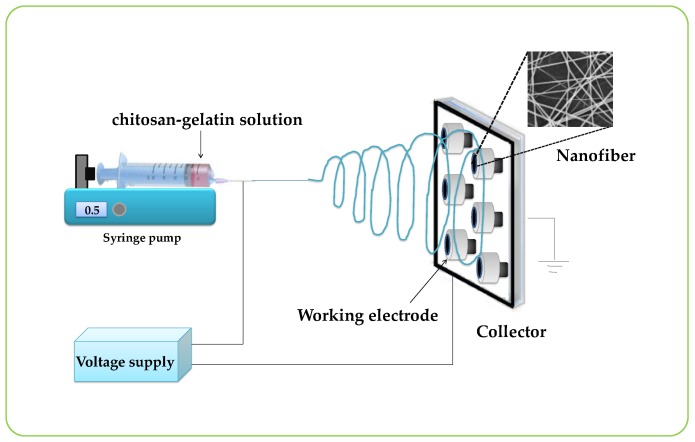
Schematic drawing of the electrospinning system.

A schematic drawing of the electrospinning setup for this work is presented in Figure 1. Typically, electrospinning was performed by a DC power supply (Gamma High Voltage Research, Ormond Beach, FL, USA) at 0–20 kV, a syringe pump (KDS100, KD Scientific Inc., Holliston, MA, USA), and a collector. The graphite electrode (diameter = 2 mm), used as a collector, was connected to the ground electrode of the power supply and was placed parallel to the needle of a syringe. The electrospinning process was carried out at room temperature. The chitosan-gelatin solution was loaded into the syringe and was ejected from the needle tip by a syringe pump. In this study, a constant flow rate of 1.6 mL/h was applied to the chitosan-gelatin solution. The nanofibers were collected on the graphite electrode surface that was placed on an aluminum foil rotating collector. The distance between the needle tip of the syringe and the collector was 20 cm. The gelatin/chitosan nanofibers-modified graphite electrode was obtained and ready to use for the enzyme immobilization step after drying.

### 2.3. Fabrication of the Enzymatic Biosensor Based on Nanofibers

The biosensor was prepared as follows: Firstly, nanofibers modified electrode was immersed into 2.5% *v/v* of glutaraldehyde for 20 min to make the covalent binding with enzyme. After washing, the electrode was immersed in HRP for 12 h to be immobilized on the nanofibers. The electrode was finally washed with phosphate buffer to remove unbound enzyme. Finally, 1% (*v/v*) nafion solution was dropped onto the electrode and dried at room temperature. Hence, the modified electrode was successfully achieved. The prepared electrodes were stored at 4 °C in a pH 7.00 phosphate buffer solution until further use.

### 2.4. Characterization of Nanofibers

The morphology and diameter of nanofibers samples were determined using scanning electron microscopy (SEM; JSM-6510, JEOL, Peabody, MA, USA). For SEM sample preparation, the sample was attached to a SEM stub and then was sputtered with a thin layer of gold. SEM was performed under an accelerating voltage of 5 kV.

### 2.5. Electrochemical Measurement

The electrochemical experiments were performed with a CHI660a electrochemical workstation (CHI, Austin, TX, USA). All experiments were carried out with a three-electrode system with an enzyme nanofibers-modified graphite electrode as the working electrode, a platinum wire as the auxiliary electrode, and an Ag/AgCl/3.0 M KCl electrode as the reference electrode. Cyclic voltammetric was performed in 10 mM K_3_Fe(CN)_6_/K_4_Fe(CN)_6_ and 10 mM KCl solution to characterize the immobilization steps on the electrode surface. Amperometric measurements were carried out under stirred phosphate buffer solution (phosphate buffer, 0.15 M, pH 6.00) by applying the potential of −0.3 V at room temperature (25 °C).

### 2.6. Optimizations of Experimental Conditions of Enzyme Biosensor

The performance of this biosensor was dependent on various factors, such as the concentration of the enzyme, the pH and concentration of phosphate buffer, and the applied potential for the detection of hydrogen peroxide. Therefore, these parameters were studied to obtain a high analytical signal.

### 2.7. Evaluation of the Nanofiber Biosensor

Under optimum conditions, the performance of the biosensor system was evaluated by studying the precision, linearity range, detection limit, storage stability, and application in real samples.

## 3. Results

### 3.1. Compositions and Characterizations of Nanofibers

**Figure 2:**
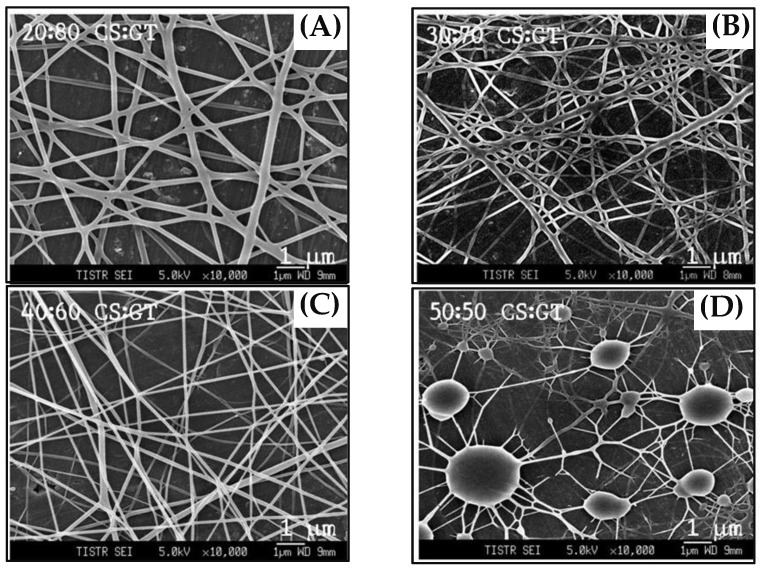
SEM micrographs of nanofibers at different ratios of chitosan and gelatin: (**A**) 20:80; (**B**) 30:70; (**C**) 40:60; and (**D**) 50:50, respectively.

Figure 1 shows the schematic of the experimental setup used for the electrospinning process. The nanofibers compositions were optimized by mixing the solution of chitosan and gelatin at different ratios to obtain the best nanofibers. Nanofibers were electrospun from pure chitosan and the other four sets of chitosan-gelatin with volume ratios of chitosan to gelatin solution of 50:50, 40:60, 30:70, and 20:80, respectively. The morphological structures of the electrospun chitosan-gelatin composite nanofibers are shown in Figure 2. The results found that pure chitosan cannot produce a continuous and uniform fiber because chitosan molecules presented many amino groups resulting in high surface charge densities of the jet. Thus, the electric field was not sufficiently strong. Charges on the droplet surface did not overcome the surface tension, causing nonuniform and discontinuous fibers [27]. To overcome this, different ratios of gelatin were added. The addition of gelatin decreased the conductivity of the solution, thus producing continuous nanofibers. Therefore, chitosan and gelatin ratios of 40 to 60 were selected. Small diameters of fibers without any beads were obtained.

### 3.2. Construction of HRP/Nanofibers/Electrode

**Figure 3:**
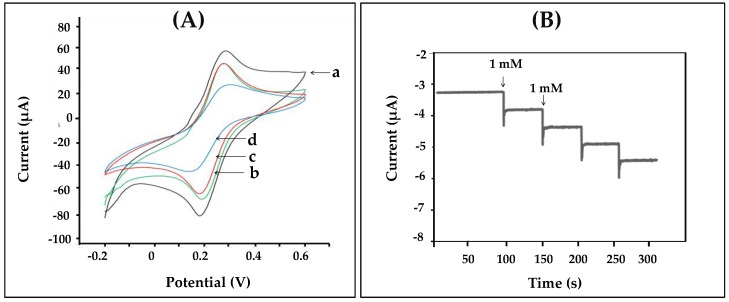
(**A**) Cyclic voltammograms obtained from different modified electrodes (a) bare, (b) nanofibers, (c) glutaraldehyde, and (d) HRP in 10 mM K_3_Fe(CN)_6_/K_4_Fe(CN)_6_ containing 0.1 M KCl at a scan rate of 50 mV s^−1^; (**B**) shows the amperometric responses obtained from nanofibers-modified electrodes to successive addition of 1 mM hydrogen peroxide in phosphate buffer at the applied potential of −0.2 V.

As shown in Figure 1, we designed the construction of the nanofiber enzyme biosensor using HRP immobilized onto a chitosan-gelatin composite nanofibers-modified electrode as a working electrode. Modified electrodes were characterized by cyclic voltammetry in 0.1 M K_3_Fe(CN)_6_/K_4_Fe(CN)_6_ at a scan rate of 50 mV/s. Figure 3A displayed the cyclic voltammogram results obtained during each step of immobilizations. The cyclic voltammogram of the bare electrode (a) showed complete oxidation and reduction peaks of $\mathrm{Fe}{\left(\mathrm{CN}\right)}_{6}^{3-/4-}$ redox. After the electrode surface was coated with chitosan-gelatin composite nanofibers (b), the anodic and cathodic peak currents decreased, as indicated that the nanofibers that were immobilized on the electrode. Then the redox peaks slightly decreased because the amine groups of chitosan and gelatin on the surface of the electrode were activated with glutaraldehyde (c). Finally, after HRP was immobilized on the surface of the electrode, and the peak current decreased greatly. These results confirmed that HRP was successfully immobilized on the nanofibers-modified electrode. To test the activity of the enzyme-modified electrode, three electrodes were connected to a potentiostat and immersed into 2 mL, 0.05 M phosphate buffer (pH 7.20). Methylene blue (MB) was used as a mediator. The reaction occurred by adding hydrogen peroxide solution to reach a final concentration 1 mM. The reaction mechanism of the hydrogen peroxide biosensor is reported as follows [27]:

HRP_(red)_ + H_2_O_2_ → HRP_(ox)_ + H_2_O (1)

HRP_(ox)_ + MB_(red)_ → HRP_(red)_ + MB_(ox)_ (2)

MB_(ox)_ + 2e^−^ → MB_(red)_ (3)

The generated current was measured by the amperometric technique. The amperometric responses of the nanofibers-modified electrode at a working potential of −0.2 V for each successive addition of various amount of hydrogen peroxide are presented in Figure 3B. It was confirmed that nanofibers were successfully used to immobilize the enzyme. In addition, the current decreased sharply to reach a steady state value (ca. 95%) in less than 20 s, when the standard of hydrogen peroxide was added to the phosphate buffer. Therefore, the enzyme-modified electrode via nanofibers showed good response to hydrogen peroxide and was related to the concentration of hydrogen peroxide in solution.

### 3.3. Effect of Electrospinning Parameters

There are many factors affecting the morphologies of electrospun nanofibers, such as solvent polarity, applied voltage, feed rate, needle diameter, and collection distance. All of thsse factors significantly affect the quality of the nanofibers. Therefore, these parameters were studied in this present work.

In order to best optimize the nanofiber, the acetic acid concentrations were varied, ranging from 1 to 90%, whereas all other parameters were kept constant. Decreasing the acetic acid concentration in the solvent increases the mean diameter of the nanofibers. An optimum size of nanofibers was achieved and 60% acetic acid was found to be most suitable for the formation of uniform nanofibers.

Once an electric field is applied, the surface of the polymer solution becomes charged. Thus, the effect of the applied electric field was studied from 15 to 20 kV. The voltage was gradually increased up to 20 kV until the liquid came out through the needle and split into a web of fibers collected on the aluminum foil. Twenty kilovolts was selected.

The flow rate of the polymer solution within the syringe is another important process parameter. The flow rate was optimized in the range of 0.8–1.6 mL/h. At a low flow rate (0.8–1.4 mL/h) the electrospun fiber is cylindrical and uniform. The results found that the continuous fibers were obtained at the flow rate of 1.6 mL/h.

The effect of the diameter of the syringe needle was investigated from 0.5–0.7 mm. The increase of the needle diameter from 0.5 to 0.6 mm significantly increased the average nanofiber diameter. The nanofiber obtained with a larger needle diameter showed a more uniform fiber size distribution. The optimum diameter of the syringe was 0.6 mm.

The distance between the collector and the tip of the syringe can also affect the fiber diameter and morphologies. Thus, the collection distance was studied by varying it from 20 to 25 cm. At a distance of 15 cm, the fiber will not have enough time to solidify before reaching the collector, whereas at a distance of 25, fiber beads can be obtained. The optimum collection distance was at 20 cm.

The optimum conditions resulting in a continuous fiber with an average diameter of 80 nm are featured in Table 1.

### 3.4. Optimized Experimental Conditions for the Hydrogen Peroxide Biosensor

The nanofiber-modified electrode was immobilized with HRP and was then applied in a biosensor for hydrogen peroxide detection. In order to obtain the highest sensitivity of the proposed biosensor system, several parameters, including enzyme concentration, applied voltage, buffer concentration, and pH of solution buffer, were studied.

**Figure 4:**
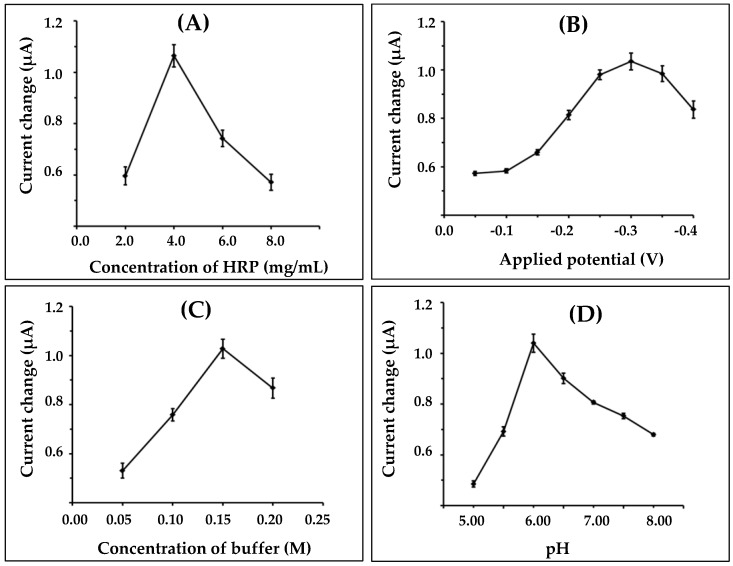
Effect of (**A**) concentration of HRP; (**B**) applied potential; (**C**) concentration of buffer; and (**D**) pH on the response current of the nanofiber electrode.

First, the immobilizations of different amounts of HRP were optimized in the range of 2.0–8.0 mg/mL. Results obtained from the experiments are shown in Figure 4A. The biosensor response was increased as the amount of HRP was increased, up to 4 mg/mL; after that it was decreased. The current response decreased probably due to the steric hindrance effect caused by a high density of immobilized enzyme on the electrode surface, and it created a difficulty in the transfer of electrons between the enzyme and the electrode surface. Consequently, in this case, 4.0 mg/mL of HRP was chosen for further optimization.

The influence of applied potential on the nanofiber-modified working electrode for amperometric measurement, from −0.05 to −0.4 Vvs. Ag/AgCl onto the enzyme biosensor system was investigated. Figure 4B shows the resulting plot of the current as a function of applied potential. It was found that the current increased with the increasing potential from −0.05 to −0.3 V, and then decreased after −0.3 V. Therefore, a potential of −0.3 V was selected as the applied potential.

The concentrations and pH of buffer, which were variable in the proposed work, could affect the enzymatic biosensor for hydrogen peroxide detection. The effect of the different buffer concentrations on the response of the nanofiber-enzyme electrode is shown in Figure 4C. The current response gradually increased as the concentration increased from 0.05 to 0.15 M. The highest response was achieved at 0.15 M. Thus, the optimal buffer concentration of 0.15 M was chosen for the supporting electrolyte.

The pH value of the buffer is another variable that affects the biosensor measurement. The pH effect on the biosensor performance was studied using a 0.15 M phosphate buffer solution, and pH varied between 5.00 and 8.00 (Figure 4D). The maximum current response was obtained at pH 6.00, which was similar to that reported previously [28].

### 3.5. Nanofiber Biosensor Performance

Under the optimized conditions, the biosensor was evaluated by studying the analytical precision, linear range, limit of detection, and storage stability.

Analytical precision was carried out by measuring the current response of the same nanofibers-enzyme electrode in the presence of 1 mM hydrogen peroxide. The average mean steady-state of the current response was 1100 nA with the relative standard deviation (RSD) of 3.3% for twelve successive assays. Therefore, the nanofiber-modified electrode showed high precision.

**Figure 5:**
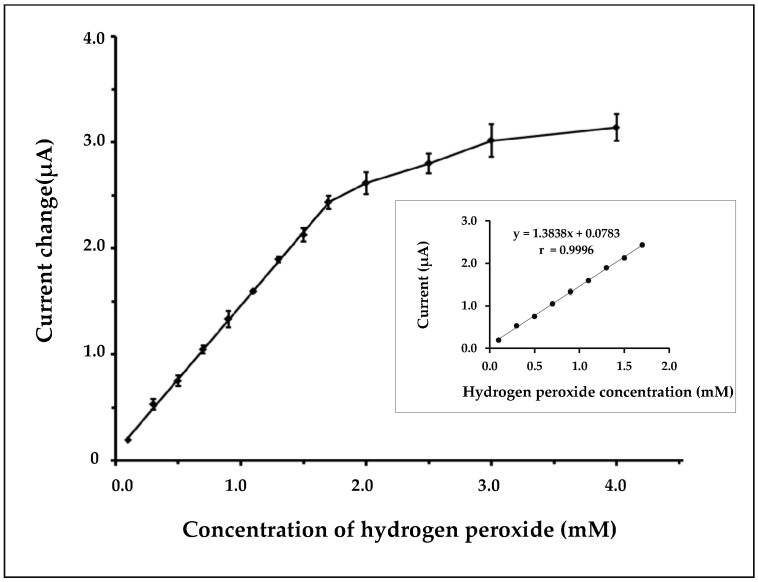
Calibration curve at different concentrations of hydrogen peroxide. The error bars show standard deviations for *n* = 3. Inset: the linear part of the calibration curve.

Figure 5 shows the resulting calibration curve for hydrogen peroxide detection over the concentration range of 0.1–4.0 mM. The linear calibration range of the nanofiber biosensor was observed from 0.1 to 1.7 mM with a correlation coefficient of 0.9996. The nanofiber biosensor showed a sensitivity of 44 µA/mM/cm^2^ and the response time of the proposed method was around 10 s. A detection limit of 0.05 mM (S/N = 3) was obtained.

The selectivity of the fabricated sensor was tested with possible interferences, such as ascorbic acid, glucose, and uric acid. It was found that that there was no significant change of the current responses generated from ascorbic acid, glucose, and uric acid compared to the response of H_2_O_2_. Therefore, the proposed sensor was selective to H_2_O_2_ detection.

Additionally, the stability of the fabricated sensor was evaluated by amperometric measurements in the presence of H_2_O_2_. No significant decrease of current was measured after the electrode was studied 20 times continuously. Additionally, the storage stability of the nanofibers enzyme-modified electrode was studied by monitoring the current response of 1 mM of hydrogen peroxide every week and it was stored at 4 °C in a refrigerator after use. The results show that there was only a 14% loss from its initial current response after two months of use, indicating that the immobilized HRP on gelatin/chitosan nanofibers possessed good bioactivity.

### 3.6. Comparison of Current Response between Nanofibers and Thin Film-Modified Electrodes

**Figure 6:**
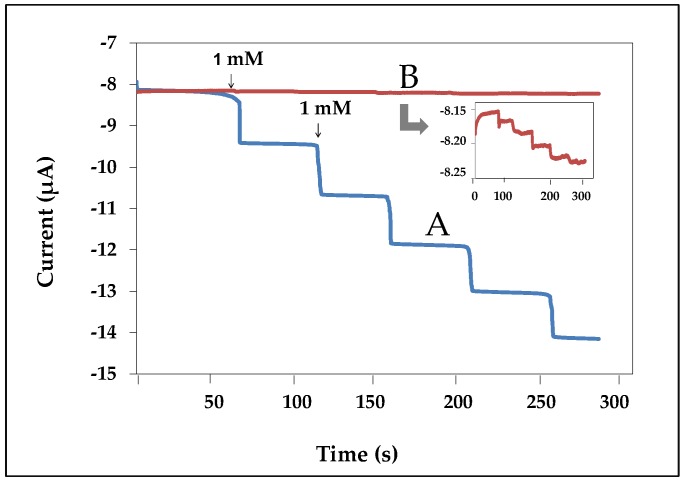
Amperometric responses obtained from nanofiber (A) and thin film (B) modified electrodes to successive addition of 1 mM hydrogen peroxide in phosphate buffer at the applied potential of −0.3 V.

The generated current was measured by the amperometric technique. Figure 6 showed the comparison current response obtained from (a) nanofiber- and (b) thin film-modified electrodes. The thin film-modified electrode was carried out by dropping a mixture solution of chitosan gelatin with volume ratios of 40:60, respectively, on the working electrode and then allowed to dry at room temperature. The thin film-modified electrode was immersed into 2.5% *v/v* of glutaraldehyde for 20 min and was immersed in HRP for 12 h. Finally, the electrode surface was cover with 1% (*v/v*) nafion solution. The results found that the nanofibers-modified electrodes provided higher response than the thin film-modified electrodes. This confirmed that nanofibers were successfully applied for use as an enzyme biosensor.

### 3.7. Application to Real Samples

To investigate the feasibility of the nanofiber biosensor system for analysis of hydrogen peroxide in different brands of disinfectant was evaluated. The dilution of samples was required with phosphate buffer in order to fit the linear range. All samples were analyzed by the proposed method and the titration method. The redox titration was used as the standard method. This method utilizes the reduction of potassium permanganate (KMnO_4_) by hydrogen peroxide in sulfuric acid. KMnO_4_ solution (0.02 M) was employed for the standard solution. The endpoint of the titration is the point at which the last drop of KMnO_4_ added to the solution causes it to turn pink. Results obtained with the proposed method were compared statistically with those obtained with the titration method (Table 2). There was no significant difference between the two methods for hydrogen peroxide detection from disinfectant samples at the 0.05 significance level. This experiment showed that the nanofiber biosensor achieved the analysis of hydrogen peroxide in real samples.

## 4. Conclusions

From the above results, we have concluded that the nanofibers-modified electrodes exhibited good efficiency for immobilization of enzyme on the electrode surface resulting in enhancing the sensitivity of biosensor system for hydrogen peroxide detection. The detection limit and sensitivity of these electrodes was found to be 0.05 mM and 44 µA/mM/cm^2^, respectively. This present work had advantages, such as easy electrode preparation, long-term stability, and good repeatability compared to other previous works [9,22]. In addition, the nanofibers coated on the surface of the electrode not only offer biocompatibility for enzyme immobilization, but are also successful in their application to real sample analysis.

## Figures and Tables

**Table 1:** Electrospinning conditions for fabrication of nanofibers.

Effecting Parameter	Parameter Studied	Optimum
Acetic concentration	1%–90%	60%
Applied electric field	15–20 kV	20 kV
Flow rate	0.8–1.6 mL/h	1.6 mL/h
Needle diameter	0.5–0.7 mm	0.6 mm
Collection distance	15–25 cm	20 cm

**Table 2:** Determination results of hydrogen peroxide in real samples.

Sample	Concentration of Hydrogen Peroxide (mM)	
	Nanofiber Biosensor	Titration Method
Sample-1	873	871
Sample-2	826	824
Sample-3	851	853

## References

[1] Justino C.I.L., Duarte A.C., Rocha-Santos T.A.P. (2016). Critical overview on the application of sensors and biosensors for clinical analysis. Trends Anal. Chem..

[2] Batra N., Tomar M., Gupt V. (2015). ZnO-CuO composite matrix based reagentless biosensor for detection of total cholesterol. Biosens. Bioelectron..

[3] Weng X., Gaur G., Neethirajan S. (2016). Rapid Detection of Food Allergens by Microfluidics ELISA-Based Optical Sensor. Biosensors.

[4] Kim G., Moon J.H., Moh C.Y., Lim J. (2015). A microfluidic nano-biosensor for the detection of pathogenic *Salmonella*. Biosens. Bioelectron..

[5] Uzunoglu A., Stanciu L.A. (2016). Novel CeO_2_-CuO-decorated enzymatic lactate biosensors operating in low oxygen environments. Anal. Chim. Acta..

[6] Tsekenis G., Filippidou M.K., Chatzipetrou M., Tsouti V., Zergioti I., Chatzandroulis S. (2015). Heavy metal ion detection using a capacitive micromechanical biosensor array for environmental monitoring. Sens. Actuators B Chem..

[7] Bahadır E.B., Sezginturk M.K. (2015). Applications of commercial biosensors in clinical, food, environmental, and biothreat/biowarfare analyses. Anal. Biochem..

[8] Thenmozhi K., Narayanan S.S. (2017). Horseradish peroxidase and toluidine blue covalently immobilized leak-free sol-gel composite biosensor for hydrogen peroxide. Mater Sci. Eng. C.

[9] Chen C., Hong X., Xu T., Chen A., Lu L., Gao Y. (2016). Hydrogen peroxide biosensor based on the immobilization of horseradish peroxidase onto a poly(aniline-co-N-methylthionine) film. Synth. Met..

[10] MahbuburRahman M., Li X., Kim J., Lim B.O., Ahammad A.J., Lee J.J. (2014). Cholesterol biosensor based on a bi-enzyme immobilized on conducting poly(thionine) film. Sens. Actuators B Chem..

[11] Bhakta S.A., Evans E., Benavidez T.E., Garcia C.D. (2015). Protein adsorption onto nanomaterials for the development of biosensors and analytical devices: A review. Anal. Chim. Acta.

[12] Wu J., Yin F. (2013). Sensitive enzymatic glucose biosensor fabricated by electrospinning composite nanofibers and electrodepositing Prussian blue film. J. Electroanal. Chem..

[13] Zhu H., Du M.L., Zhang M., Wang P., Bao S.Y., Wang L.N., Fu Y.Q., Yao J.M. (2013). Facile fabrication of, AgNPs/(PVA/PEI) nanofibers: High electrochemical efficiency and durability for biosensors. Biosens. Bioelectron..

[14] Wang Z.G., Wan L.S., Liu Z.M., Huang X.J., Xu Z.K. (2009). Enzyme immobilization on electrospun polymer nanofibers: An overview. J. Mol. Catal. B.

[15] Jiang S., Hou H., Greiner A., Agarwal S. (2012). Tough and Transparent Nylon-6 Electrospun Nanofiber Reinforced Melamine–Formaldehyde Composites. ACS Appl. Mater. Interfaces.

[16] Shin Y.J., Kameoka J. (2012). Amperometric cholesterol biosensor using layer-by-layer adsorption technique onto electrospun polyaniline nanofibers. J. Ind. Eng. Chem..

[17] Arvand M., Ghodsi N. (2014). Electrospun TiO_2_ nanofiber/graphite oxide modified electrode for electrochemical detection of l-DOPA in human cerebrospinal fluid. Sens. Actuators B Chem..

[18] Xin M., Zhang X., Yang J., Chen M., Ma X., Liu J. (2014). Preparation and electrochemical sensing application of electrospun porous organosilica fibers. Electrochem. Commun..

[19] Kai D., Liow S.S., Loh X.J. (2014). Biodegradable polymers for electrospinning: Towards biomedical applications. J. Loh. Mater. Sci. Eng. C.

[20] Mehrasa M., Asadollahi M.A., Ghaedi K., Salehi H., Arpanaei A. (2015). Electrospun aligned PLGA and PLGA/gelatin nanofibers embedded with silica nanoparticles for tissue engineering. Int. J. Biol. Macromol..

[21] Huang C.H., Chi C.Y., Chen Y.S., Chen K.Y., Chen P.L., Yao C.H. (2012). Evaluation of proanthocyanidin-crosslinked electrospun gelatin nanofibers for drug delivering system. Mater. Sci. Eng. C.

[22] Du Z., Li C., Li L., Zhang M., Xu S., Wang T. (2009). Simple fabrication of a sensitive hydrogen peroxide biosensor using enzymes immobilized in processable polyaniline nanofibers/chitosan film. Mater. Sci. Eng. C.

[23] Tiwari1 A., Shukla S.K. (2009). Chitosan-g-polyaniline: A creatine amidinohydrolase immobilization matrix for creatine biosensor. Express Polym. Lett..

[24] Wen Y., Wen W., Zhang X., Wang S. (2016). Highly sensitive amperometric biosensor based on electrochemically-reduced graphene oxide-chitosan/hemoglobin nanocomposite for nitromethane determination. Biosens. Bioelectron..

[25] Zhang Y., Liu Y., Chu Z., Shi L., Jin W. (2013). Amperometric glucose biosensor based on direct assembly of Prussian blue film with ionic liquid-chitosan matrix assisted enzyme immobilization. Sens. Actuators B Chem..

[26] Devi R., Pundir C.S. (2014). Construction and application of an amperometric uric acid biosensor based on covalent immobilization of uricase on iron oxide nanoparticles/chitosan-g-polyaniline composite film electrodeposited on Pt electrode. Sens. Actuators B Chem..

[27] Sun K., Li Z.H. (2011). Preparations, properties and applications of chitosan based nanofibers fabricated by electrospinning. Express Polym. Lett..

[28] Lin X.Q., Chen J., Chen Z.H. (2000). Amperometric biosensor for hydrogen peroxide based on immobilization of horseradish peroxidase on methylene blue modified graphite electrode. Electroanalysis.

